# Investigating pre-hospital troponin testing in the diagnosis of acute myocardial infarction: a systematic review

**DOI:** 10.29045/14784726.2025.9.10.2.17

**Published:** 2025-09-01

**Authors:** Jenny Alexanders, Sam Mcpherson, Andrew Letheren, Robert Oxley, Joe Saunders

**Affiliations:** Teesside University 0000-0001-5519-3311; Nuffield Health; Nuffield Health; Teesside University; Teesside University

**Keywords:** emergency services, myocardial infarction, point-of-care, pre-hospital, troponin

## Abstract

**Introduction:**

Chest pain is one of the most common conditions presenting to emergency departments (EDs), with over 1.3 million attendances according to 2022/2023 UK data. The diagnosis of a myocardial infarction (MI) in pre-hospital settings is challenging, relying on careful history taking and electrocardiograms. Patients with ST elevation are transferred to hospitals with primary percutaneous coronary intervention (PPCI) facilities, whereas those without ST elevation are taken to local EDs for further work-up, including troponin testing. Point-of-care (POC) troponin tests are now available in the field, presenting the potential to diagnose MI in patients without ST elevation. This systematic review aims to investigate and determine whether POC troponin devices are effective in detecting patients with acute MI in the pre-hospital setting.

**Methods:**

This systematic review was performed in accordance with the PRISMA guidelines. A comprehensive search of the literature was created using the following databases: CINAHL, MEDLINE, EMBASE and DISCOVERY. Basic and advanced search strategies were used to identify papers using POC troponin testing in patients presenting in the pre-hospital setting with chest pain.

**Results:**

A total of five articles were identified demonstrating that a variety of pre-hospital POC troponin testing devices can detect MI in patients presenting with chest pain. One limitation of POC testing was that some patients with negative troponin results in the pre-hospital setting were confirmed to have MI in the hospital setting.

**Conclusion:**

Using POC troponin raises the possibility of detecting an MI, but cannot definitively exclude an MI. Further research is required to investigate the reliability of POC troponin testing in cohorts without ST elevation in the pre-hospital environment, which may expedite transfer to specialised PPCI hospitals over a local ED. The outcome of further research has the potential to positively impact outcomes of patients suffering with MI and to yield financial benefits via faster, more effective treatment.

## Introduction

Cardiovascular disease, particularly of a cardiac origin, is one of the leading causes of death in the developed world (Martín-Rodríguez, 2021), with chest pain accounting for one in six ambulance transfers and one in 20 walk-ins to emergency departments (EDs) (Best, 2017). Around 2–4% of patients presenting with chest pain will be diagnosed with a myocardial infarction (MI) (Andersson et al., 2015). Due to limited diagnostic tests in the pre-hospital environment, the diagnosis of MI in the field can be difficult (Johannessen et al., 2021). Clinicians are often reliant on history taking and electrocardiogram (ECG) results (Bond, 2022), including ST-segment elevation (Cooper et al., 2021). In the United Kingdom, ambulance services use clinical diagnostic criteria to transport patients with ST-segment elevation (STEMI) directly to hospitals for specialist cardiac catheterisation treatment (Cooper et al., 2021), including treatment via primary percutaneous coronary intervention (PPCI) (Tough, 2006). Most patients presenting with chest pain in the pre-hospital setting will not have STEMI on an ECG and are consequently transported to a hospital without PPCI facilities (Cooper et al., 2021).

The National Institute for Health and Care Excellence (NICE) guidelines advocate that patients presenting at the ED with chest pain of a suspected cardiac origin, who are seen to be of moderate to high risk of MI, should undergo a high sensitivity troponin test to rule out a non-ST-elevated MI (NSTEMI) (National Institute for Health and Care Excellence (NICE), 2016). The gold standard biochemical test in the diagnosis of MI is the measurement of cardiac troponin (Aldous et al., 2012); however, MI is not the only cause of an acutely elevated troponin level (Long et al., 2020). Within an ED a variety of risk stratification tools exists that can identify patients who are at high risk of a cardiac event, or at low risk and therefore suitable for discharge (Cooper et al., 2021).

Troponin is part of a group of structural proteins involved in the regulation of striated and cardiac muscle contraction, with cardiac troponin released after acute MI (Zoltani, 2014). An elevated troponin is quantified as being above the 99th percentile of the reference population and forms the decision level for the diagnosis of an MI (Thygesen et al., 2007). Historically, elevated troponin would be detected within 3–4 hours of myocardial injury, although with recent advances in sensitivity tests, elevation can now be detected much earlier, including patients in acute MI (Chung & Brown, 2018).

Standard practice involves sending troponin blood tests to a hospital laboratory. With the availability of point-of-care (POC) handheld devices, rapid troponin measurements can be undertaken at the bedside (Williams, 2022). These devices require blood to be drawn from an intravenous cannula then placed into the POC device, providing an accurate reading of the troponin levels within 8–10 minutes (van Dongen et al., 2021). These patient-side devices may have a valuable role in the pre-hospital environment (Williams, 2022), alongside reducing costs to healthcare providers, as fewer patients experiencing chest pain are referred to hospital (Kip et al., 2017).

The aim of this systematic review is to investigate and determine whether POC troponin devices are effective in detecting patients with acute MI in the pre-hospital setting.

## Methods

### Search strategy

This systematic review was performed in accordance with PRISMA guidelines (Page et al., 2021).

The PICO framework (Huang et al., 2006) was implemented to direct the research question as follows: population = patients presenting with chest pain in the pre-hospital environment, intervention = POC troponin devices, comparator = centres without POC troponin testing, outcome = detection of acute MI.

CINAHL, MEDLINE, EMBASE and DISCOVERY were systematically searched in January 2023 using the following criteria:

Troponin (AND) MI (OR) Myocardial Infarction (OR) Heart Attack (OR) non elevated myocardial infarction (OR) NSTEMI (OR) ST elevated myocardial infarction (OR) acute coronary syndrome (OR) ACS (AND) Pre hospital (OR) prehospital (OR) Out of hospital (AND) Paramedic (OR) Emergency medical services (OR) EMS (OR) Ambulance Clinician.

Additional search strategies were applied including any grey literature, hand searches of published systematic reviews and unpublished work to ensure all relevant literature was included.

### Study selection

Eligibility/inclusion criteria for this study included: adults (18 years and over); patients presenting with chest pain in the pre-hospital environment; paramedics, ambulance clinicians or emergency medical services (EMS); written in English; and involving a POC troponin test. Exclusion criteria included: involving other healthcare professionals to those listed, investigating different outcomes to POC troponin tests and differing from the pre-hospital environment.

Two researchers (JS and JA) independently screened the identified articles and then compared their findings. Title and abstract were initially assessed against the inclusion and exclusion criteria. The second sifting process involved reading the full article and assessing it against the inclusion and exclusion criteria. Both researchers completed an exclusion table of articles at this stage.

### Study appraisal

The eligible studies were assessed using the Critical Appraisal Skills Programme (CASP) (CASP, 2023). The results of the CASP screening are presented in Supplementary 1.

## Results

**Figure 1:**
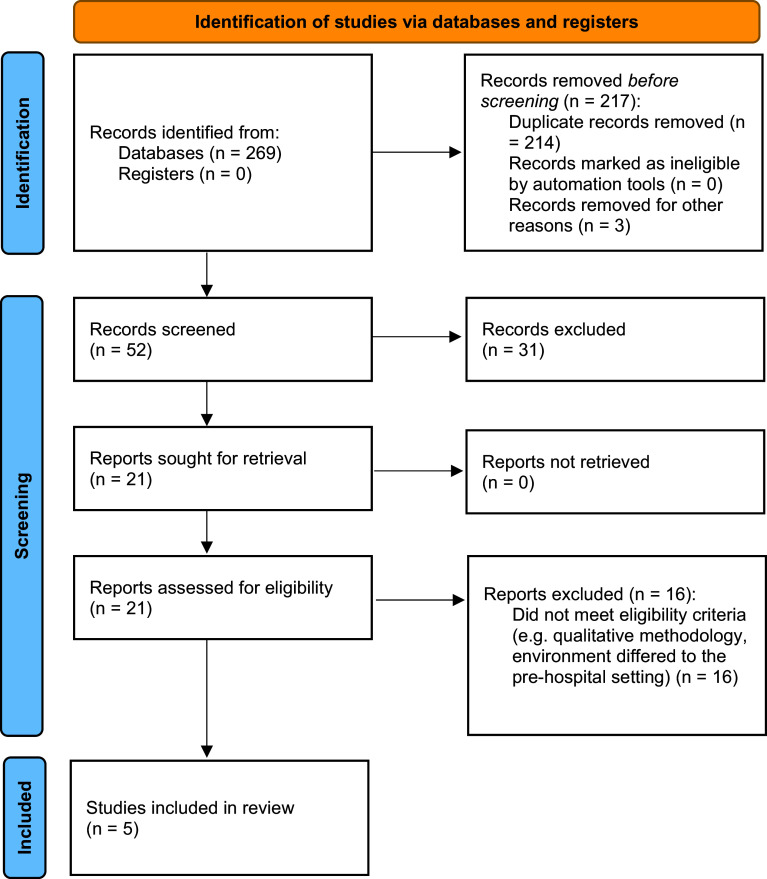
Figure 1. PRISMA flow chart detailing the study selection process.

The PRISMA guidelines were used for this systematic review, and a total of 269 articles were identified on completion of the search and screening process. Following screening, five articles were found to meet the criteria for inclusion in the final synthesis, detailed in Figure 1. The results from the search are shown in Table 1.

**Table 1.** Results table of the included studies.

Study	Design	Participants/setting	Intervention	Outcomes	Results
Rasmussen et al. (2019)	Observational follow-up study	18,712 (59% male, 52–79 years) patients presenting to EMS in Denmark with symptoms of an MI.	POC troponin via Roche Cobas h232	Diagnosis of MI when admitted to hospital	16,652 cases of POC troponin <50 ng/l, 1221 (7.3%) diagnosed with MI. 2150 POC troponin of >50 ng/l, of which 966 (44.9%) diagnosed with MI.
Schuchert et al. (1999)	Cohort study	158 patients assessed by ambulance crew with acute chest pain in Hamburg, Germany (87 male, 55–85 years).	POC troponin test	+ve/-ve troponin. Diagnosis of MI or death within the following 6 months	40 patients diagnosed with MI, of which 7 had a positive pre-hospital troponin test. 11 patients had positive pre-hospital troponin, 8 died either during hospital admission or on follow-up.
Stengaard et al. (2013)	Observational prospective study	985 patients (583 male, 59–71 years) with MI symptoms assessed by paramedics in Denmark.	POC troponin via Roche Cobas h232	Unstable angina or acute MI	31/985 were diagnosed with unstable angina, 200/985 with MI; 754/985 had no cardiac event. Further follow-up of 924 patients was undertaken. 817/924 patients with a pre-hospital troponin of <50 ng/l. 14.5% were diagnosed with an MI. 107/924 with a POC troponin of >50 ng/l. 68% were diagnosed with MI.
Stopyra et al. (2020)	Cohort study	506 patients above the age of 21, with acute non-traumatic chest pain, presenting to EMS in North Carolina, USA (53.2% female, 46–74 years).	POC troponin via i-STAT device	Diagnosis of acute MI	421 patients had a successful pre-hospital troponin test. Of these 21/421 had elevated troponin, with 85.7% diagnosed with MI. 400 patients with a negative pre-hospital troponin, 12.5% were diagnosed with MI in hospital.
Sørensen et al. (2021)	Cohort study	4905 patients presenting to EMS in Denmark with suspected acute coronary syndrome (60% male, 55–79 years).	POC troponin via quantitative troponin test kit and ECG	Diagnosis of acute MI	928/4905 had successful pre-hospital troponin tests, 3947 had only ECG recorded. 69/928 had elevated troponin, 91% diagnosed with MI. 859/928 had negative troponin group, 16.2% diagnosed with an MI.

The included studies demonstrated that pre-hospital troponin testing may be useful in detecting MI in patients presenting with chest pain, using a variety of devices and methods. However, in some incidences, where the POC troponin test was negative the patients were later diagnosed with an MI in a hospital setting.

Both Rasmussen et al. (2019) and Stengaard et al. (2013) used the Roche Cobas h232, a quantitative POC device giving results of either greater or less than 50 nanograms per litre (ng/l). The patient group found to have a result >50 ng/l and diagnosed with MI ranged between 44.9% and 68.2% (Rasmussen et al., 2019; Stengaard et al., 2013). Of those patients with a result <50 ng/l, between 7.3% and 14.5% were diagnosed with MI (Rasmussen et al., 2019; Stengaard et al., 2013). In both these studies, a much larger proportion of patients with a pre-hospital troponin of >50 ng/l were diagnosed with an MI than those <50 ng/l.

Sørensen et al. (2021) used an undisclosed quantitative method of pre-hospital troponin testing. They observed that, of the patients with a positive pre-hospital troponin (>0.1 nanograms per millilitre (ng/ml)), 91.3% were diagnosed with an MI. Of the group of patients who had a negative pre-hospital troponin test (<0.1 ng/ml), only 16.2% were diagnosed with an MI.

Stopyra et al. (2020) used the i-STAT POC device, defining a positive result as a troponin >0.08 ng/ml. Of the patients presenting with a positive pre-hospital result, 85.7% were diagnosed with an MI, whereas of those with a negative pre-hospital result (<0.08 ng/ml), 12.5% were later diagnosed with an MI.

Schuchert et al. (1999) used a rapid troponin test that gave either a positive or negative result at the scene of the emergency. Of the 158 patients tested, 40 (25.3%) were diagnosed with an MI and, of these, seven had a positive pre-hospital troponin test. Schuchert et al. (1999) performed a six-month follow-up and found that of the 11 patients with a positive troponin, eight had died within six months, five of which died in hospital and a further three at a later date.

## Discussion

The aim of this systematic review was to investigate and determine whether POC troponin devices are effective in detecting patients with an acute MI in the pre-hospital setting. All the included studies within this review appear to show that, although a POC test can be utilised to give an indication of an MI, it cannot exclude MI in the pre-hospital environment. This is corroborated by Svensson et al. (2003), who found that approximately 20% of patients were given a diagnosis of MI when elevated biochemical markers were found on a POC troponin test. These findings highlight the potential for a POC test to predict an MI within the pre-hospital environment; however, with only 20% of patients showing elevated biochemical markers at the time of pre-hospital testing, versus the 167 patients that were given a final diagnosis of MI, the validity of the results is uncertain (Govani & Higgins, 2012).

Within the ED, a variety of risk scores and pathways are used to identify how likely a patient is to experience an MI. The HEART score is one example of a risk score and incorporates the patient’s history, ECG, age, co-morbidities and a troponin measurement. It provides a measure of whether a patient is at low (score of 0–2), intermediate (score of 4–6) or high risk (score >7) of MI (Cooper et al., 2021). In a variety of studies, there have been attempts to use this score in the pre-hospital environment, with the help of POC troponin tests to understand whether the score could be used to identify patients at a high risk of acute MI (Cooper et al., 2021; Stopyra et al., 2020). One of these studies, by Cooper et al. (2021), compared the HEART score with the HEAR score (the HEART score without the troponin test), following patients for 30 days to determine whether any of them had any major adverse cardiac events (MACEs). The results reported that the risk of MACEs in patients was similar, irrespective of whether a troponin test was performed, and that both the HEAR or HEART scores do not safely rule out the risk of any major cardiac events from occurring. Correspondingly, one study used a simplified HEART score in the pre-hospital environment, enabling a quicker judgement to be made by the clinician on scene (Stopyra et al., 2020). If the POC troponin was positive, the patient was classified as being high risk, whereas if the POC troponin was negative, the patient was classified as either moderate or low risk depending on the remaining components of the HEART score (Stopyra et al., 2020). The results of this study revealed that patients with a positive pre-hospital troponin test (high risk) were highly likely to have a cardiac event within the next 30 days, whereas patients with a negative pre-hospital troponin test (low and moderate risk) had fewer MACEs within 30 days (Stopyra et al., 2020).

Both Cooper et al. (2021) and Stopyra et al. (2020) showed with large cohort studies that a pre-hospital HEART score can be used to predict MACEs within the next 30 days but that it is unable to exclude MACEs. Furthermore, despite Stopyra et al. (2020) reporting that a positive pre-hospital troponin test may reveal a higher risk of MACEs, Cooper et al. (2021) had both a larger number of participants and easier transferability of the results to UK practice. Stopyra et al. (2020) performed their work in America, which may be subject to differences in healthcare provision and delivery (Raby & McNaughton, 2021).

Van Dongen et al. (2021) compared the HEART score of patients in the pre-hospital environment and in the ED, observing that 75% of cases matched. Where the scores did not match it was believed that paramedics were overestimating components of the HEART score, including the history and ECG results (Van Dongen et al., 2021). Van Dongen et al. (2021) proposed that more objective scoring criteria and further training for the paramedics may improve the accuracy of the risk classification for patients using this method.

Johannessen et al. (2021) concluded that a single troponin test performed better than the HEART score in ruling out an MI, with no patients being classified as low risk diagnosed with MI in the troponin group. Of the patients classified as low risk via the HEART score, five went on to be diagnosed with an MI (Johannessen et al., 2021). This appears to contradict the other reviewed studies, as it demonstrates that a single troponin test can be used to rule out the possibility of a patient being diagnosed with an acute MI. Cardiac troponins (cTnT and cTnI) are proteins that regulate calcium-mediated interactions and their serum levels are elevated with the presence of damaged heart tissue. Evidence over the years has debated the sensitivity of detecting cTnT and cTnI and, according to Tiwari and Aw (2024), Hs-cTn assays are more sensitive than traditional cardiac troponin assays due to detecting smaller amounts of cardiac troponin in the blood. Venturini et al. (2013) assessed the reliability of POC troponin by comparing POC troponin sample results in both the ED setting and a moving ambulance. The results revealed little difference, with POC providing similar results in both settings (intraclass correlation coefficient = 0.997 with 95% CI 0.994–0.998). Although research by Venturini et al. (2013) has the potential to demonstrate that POC troponin devices are effective and reliable in the pre-hospital environment, caution should be noted given the sample size of 39 (Gumpili & Das, 2022). Additionally, the samples were not performed within the pre-hospital environment, limiting the transferability of the results to this research (Raby & McNaughton, 2021).

Nilsson et al. (2014) investigated the cost implications of introducing a POC troponin test into a primary care setting in Sweden. They compared referrals to seven primary health centres with symptoms of a cardiac complaint, of which three centres had access to troponin testing. Of the centres using the POC troponin, a greater proportion of patients were discharged home, although two patients went on to have a cardiac event within the next 30 days. In comparison with the centres without POC troponin testing, a larger proportion were referred to hospital and none of the patients discharged home were found to have a cardiac event within 30 days.

Although the results revealed a cost-reducing outcome by lessening hospital referrals, there is the chance of misdiagnosing a small number of patients, potentially putting them at risk of a major cardiac event. Stopyra et al. (2020) suggest that patients with elevated troponin could be diverted to a cardiac centre with cardiac catheterisation facilities over a local ED, which may have an effect on overall ED admission. Williams (2022) also argues that any initial financial investment in POC technology would eventually be recovered, due to the potential reduction in hospital stays and fewer secondary ambulance transfers to PPCI centres from ED.

### Limitations

This review was undertaken using a comprehensive search strategy in accordance with PRISMA guidelines, revealing five cohort studies with differing POC troponin devices. There were some limitations of this review. The studies that were included in the results were only from three countries (Denmark, Germany and America). To potentially inform policy and practice, a much wider spread of geographical regions/countries should have been included. The databases searched contained paramedic-related articles; however, including other databases, such as AMED and Cochrane Library, may have identified more articles for the results.

## Conclusion

The findings from this systematic review highlight that it is possible to detect an MI with pre-hospital POC troponin testing, but it is not possible to exclude an MI. POC troponin testing has the potential to divert patients with raised troponin levels to specialised cardiac hospitals rather than local ED. Despite the initial cost implications of providing POC troponin testing in the pre-hospital environment, this may be offset by the reduced secondary ambulance transfers and hospital stays.

Not enough evidence is currently available to widely implement this process into current practice, and further research such as a randomised control trial in this area is needed.

## Author contributions

JA performed an initial scoping search of the literature (separate to JS) to strengthen the justification for the research title, performed the first and second screening of all articles (independent of JS) and assisted with undertaking the critical appraisal of included articles.

SM assisted with the interpretation of the data, assisted with the writing of the background and conclusion sections, reviewed the entire manuscript and updated evidence and the criticality of the work. AL reviewed the accuracy of the data within the results, and assisted with the narrative synthesis, drafting and writing of the discussion section. RO assisted with undertaking critical appraisal, reviewed the search process (reviewing the articles that were included and excluded by JA and JS) to ensure that the inclusion and exclusion criteria had been followed by both researchers and reviewed all excluded articles to confirm these met the exclusion criteria. JS performed a scoping search of the literature to assist with the conception and research topic and was responsible for conception of the PICO, search terms/phrases, production of inclusion/exclusion criteria and performing searches (including first and second stage screening). JA acts as the guarantor for this article.

## Conflict of interest

None declared.

## Ethics

Not required.

## Funding

None.

## Statement of generative AI in scientific writing

The authors did not use a generative artificial intelligence (AI) tool or service to assist with preparation or editing of this work.
